# Purpose definition as a crucial step for determining the legal basis under the GDPR: implications for scientific research

**DOI:** 10.1093/jlb/lsae001

**Published:** 2024-02-01

**Authors:** Regina Becker, Davit Chokoshvili, Adrian Thorogood, Edward S Dove, Fruzsina Molnár-Gábor, Alexandra Ziaka, Olga Tzortzatou-Nanopoulou, Giovanni Comandè

**Affiliations:** Luxembourg National Data Service, L-4362 Esch-sur-Alzette, Luxembourg; Luxembourg National Data Service, L-4362 Esch-sur-Alzette, Luxembourg; Terry Fox Research Institute, V5Z 1L3 Vancouver, Canada; School of Law, University of Edinburgh, EH8 9YL, Edinburgh, UK; Faculty of Law, Heidelberg University, 69117, Heidelberg, Germany; Tilburg Institute for Law, Technology & Society (TILT), Tilburg University, Tilburg 5037 DB, Netherlands; MPLegal, Athens 15231, Greece; Legal Department, Biomedical Research Foundation of the Academy of Athens, Athens 11527, Greece; Sant’Anna School of Advanced Studies, 56127, Pisa, Italy

**Keywords:** data protection, GDPR, lawfulness, legal basis, purpose specification, scientific research

## Abstract

The General Data Protection Regulation (GDPR) of the European Union, which became applicable in 2018, contains a new accountability principle. Under this principle, controllers (ie parties determining the purposes and the means of the processing of personal data) are responsible for ensuring and demonstrating the overall compliance with the GDPR. However, interpretive uncertainties of the GDPR mean that controllers must exercise considerable judgement in designing and implementing an appropriate compliance strategy, making GDPR compliance both complex and resource-intensive. In this article, we provide conceptual clarity around GDPR compliance with respect to one core aspect of the law: the determination and relevance of the purpose of personal data processing. We derive from the GDPR’s text concrete requirements for purpose specification, which we subsequently apply to the area of secondary use of personal data for scientific research. We offer guidance for correctly specifying purposes of data processing under different research scenarios. To illustrate the practical necessity of purpose specification for GDPR compliance, we then show how our proposed approach can enable controllers to meet their compliance obligations, using the example of the overarching GDPR principle of lawfulness to highlight the relevance of purpose specification for the identification of a suitable legal basis.

## I. INTRODUCTION

The General Data Protection Regulation of the European Union (EU), henceforth the GDPR, became applicable in all EU Member States in May 2018, and also applies to all countries in the European Economic Area (the EEA), being Iceland, Norway, and Liechtenstein. The GDPR replaced the previous Data Protection Directive 95/46/EC (DPD), and doing so, established a more comprehensive and up-to-date legal framework around data protection. The overarching objective of the GDPR is to ensure that the fundamental rights and freedoms of natural persons, ‘in particular their right to the protection of personal data’, are respected.^1^ At the same time, the GDPR seeks to facilitate the use of personal data pertaining to natural persons in a manner that benefits broader society, achieving greater economic and social integration goals of the EU.^2^ This dual objective of the GDPR is reaffirmed in Article 1, which states that ‘[the] free movement of personal data within the Union shall be neither restricted nor prohibited for reasons connected with the protection of natural persons with regard to the processing of personal data’.^3^

The GDPR departs from its predecessor DPD in various substantive ways. One such departure is reflected in the fundamental approach to assessing GDPR compliance among the parties engaged in processing of personal data. Most notably, the GDPR redefines the compliance obligations of data controllers, that is, parties determining the purposes and the essential means (or the ‘why’ and the ‘how’) of the processing of personal data.^4^ Whereas under the DPD, controllers already had the responsibility to design processing in a compliant manner, the GDPR introduces the accountability principle, which additionally requires the controllers to also be able to demonstrate that processing complies with all the principles of the GDPR under Article 5(1).^5^ By emphasizing the controller’s accountability, the GDPR effectively promotes a more deliberative approach to designing data processing operations undertaken by controllers.^6^ The controllers are required to exercise considerable judgement in ascertaining that not only have they taken adequate steps to comply with the principles of the GDPR, but they have also implemented appropriate procedural and documentation strategies whereby this compliance can be demonstrated. The purpose serves an important point of departure in the determining and justification of what is necessary in terms of processing, and thus the demonstrated compliance. At the same time, the abstraction of terms in the GDPR and the lack of translation of these terms into concrete instructions (including, as will be seen, concrete guidance to date from the European Data Protection Board, or EDPB, an independent body tasked with ensuring consistent application of the GDPR and promoting cooperation among the EU’s data protection authorities) require a good deal of rigorous interpretation and create an inherent legal uncertainty.

The resultant legal uncertainty makes it challenging for controllers to ensure that the processing of personal data they undertake remains compliant with the GDPR. In this regard, GDPR compliance has been described as both broad and resource-intensive, requiring not only significant legal expertise, but also the resources to implement appropriate technical and organizational measures for ensuring compliance.^7^ Moreover, owing to the legal uncertainties of the GDPR, the risk of non-compliance and the resultant repercussions, including potential fines imposed by data protection authorities, are difficult to fully eliminate, effectively making processing of personal data an inherently liability-prone activity under the current regulatory regime.^8^

One area where the GDPR compliance challenges, due to legal uncertainties, have been associated with negative societal consequences is scientific research. Organizations undertaking processing of personal data for scientific research are often public universities; a fair number may be under-funded and lack adequate resources and legal expertise to ensure GDPR compliance.^9^ The GDPR compliance challenges faced by universities and research organizations (eg charities, non-profit organizations, start-up companies) are further amplified by the fact that scientific research is a collaborative endeavor, often requiring data sharing among institutions. In practice, this means that organizations participating in collaborative research projects must assess their compliance obligations in the context of the overall data processing, which additionally requires considerable coordination to achieve GDPR compliance across all research collaborators.^10^ The outcome is that research organizations are often discouraged from engaging in research collaborations, as they tend to perceive data sharing under the GDPR as inherently more risky and liable to fines, which may translate into foregone societal benefits in the EU.^11^ Interestingly, this happens despite the research-friendly legal regime created by the GDPR, whereby scientific research is often deemed to have a privileged position.^12^

In this article, we set out to describe an approach aimed at guiding researchers’ GDPR compliance efforts. The cornerstone of this proposed approach is correct specification of the purposes of processing personal data: that is, explicitly delineating and describing, in an appropriate level of detail, the purposes for which the controller intends to process personal data. We start by outlining the relevance of purpose specification for GDPR compliance, followed by proposing a general framework for determining purposes, which we derive from the provisions of the GDPR. Subsequently, in applying this general framework to scientific research and secondary use of data, we highlight the unique challenges to purpose specification in the context of secondary use of data for scientific research and provide guidance for correctly specifying purposes for the intended processing under different research scenarios. To conclude, we illustrate how for controllers, purpose specification can act as a critical enabler for meeting compliance obligations, using the example of compliance with the overarching GDPR principle of lawfulness.

## II. GDPR COMPLIANCE FRAMEWORK: FOCUS ON SPECIFIED PURPOSES

Defining purposes of processing personal data is a crucial component for the controller’s overall GDPR compliance. Already in the core paragraph, Article 5(1) of the GDPR, which lays down the data protection principles, four out of the six subparagraphs explicitly mention the purpose(s) of processing personal data. The crucial importance of purpose(s) is subsequently reinforced throughout Chapters III and IV of the GDPR, where the majority of the articles also refer at least in part to the purpose(s) of the processing.

In terms of Article 5 principles of the GDPR, purpose specification is most directly linked to the principle of purpose limitation, according to which personal data shall be ‘collected for specified, explicit and legitimate purposes and not further processed in a manner that is incompatible with those purposes’.^13^ When viewed through the lens of the purpose limitation principle, purpose specification can be seen as the first step in a three-step compliance process. First, the controller must clearly specify and delineate all distinct purposes for which processing of personal data is undertaken.^14^ This, in the second step, will allow the controller to determine whether or not further processing, within the meaning of Article 5(1)(b) of the GDPR, also takes place.^15^ Third, assuming further processing does indeed occur under the envisaged set-up, the controller must then assess whether this further processing can be deemed compatible with the initial purpose. Thus, through purpose specification, followed by the compatibility assessment (if applicable), the controller is enabled to demonstrate its compliance with the purpose limitation principle.^16^

However, purpose specification is also central to controllers’ compliance with other principles of the GDPR. For example, with respect to the principle of lawfulness, the EDPB states in their Guidelines 4/2019 in paragraph 68 that ‘the appropriate legal basis must be clearly connected to the specific purpose of processing’.^17^ The EDPB also stresses that in identifying the lawful basis for the processing, the ‘starting point is to identify the purpose for the processing’.^18^ As we will show in the subsequent sections, such analysis is particularly important in the context of scientific research, where the GDPR legal basis, within the meaning of Article 6(1) of the Regulation, is often (but not invariably) consent in many EU countries.^19^ Where researchers rely on a data subject’s consent as the GDPR legal basis for processing personal data, they must pay particular attention to ensure that they have correctly specified the purposes of processing. Consent is directly related to the purpose and where purpose specification does not comply with GDPR requirements, consent as a legal basis for the processing becomes invalid and the processing unlawful.

Cumulative requirements are created where the tripartite principle of lawfulness, fairness, and transparency under Article 5(1)(a) is considered through the lens of both purpose and legal basis. For example, the applicability of some of the data subject rights (under Chapter III of the GDPR) depends on the selected Article 6(1) legal basis, whereas the transparency principle requires controllers to provide appropriate information to data subjects regarding both the purpose(s) and the associated legal basis, as elaborated in Articles 13 and 14 of the GDPR.^20^ Similarly, a clearly specified purpose can help controllers determine what types of data they need to process in order to accomplish the purpose, alongside the required duration of processing, thus potentially enabling compliance with the GDPR Article 5(1) principles of data minimization and storage limitation, respectively.^21^ Purpose specification is also relevant for the accuracy principle, which explicitly states under Article 5(1)(d) that controllers, ‘having regard to the purposes for which [personal data] are processed’, must take every reasonable step to ensure that the data are accurate and up to date.^22^ The relevance of purposes for which data are processed in relation to the principle of accuracy is also stressed by the EDPB. According to the EDPB, ‘[each] personal data element should be as accurate as necessary for the specified purposes’.^23^ Finally, the dual principle of integrity and confidentiality^24^ largely concerns the conditions under which personal data should be stored, including choosing appropriate security and other technical measures, as well as determining who is authorized to access the data. Once again, defining these conditions requires a clear understanding of the specific purposes for which the personal data are processed.

Beyond the overarching Article 5(1) GDPR principles, the relevance of purpose specification for GDPR compliance can be further highlighted in the context of a controller’s concrete procedural and accountability obligations. For example, purpose specification by the controller is directly linked to the controller’s obligation to maintain a Record of data Processing Activities (RoPA) that are taking place under the controller’s responsibility.^25^ Paragraph (b) of Article 30 explicitly mandates that each entry on the RoPA must be accompanied by the description of the purposes for which the processing is carried out. This is a logical requirement, since the nature of processing operations is dictated by the purposes for which the controller intends to process the data, as will be further discussed in the subsequent sections of this article. In other words, purpose specification will typically precede, and directly influence, the design of processing operations. However, in some cases, purpose specification and design of processing operations can also be done iteratively: the initial formulation of a purpose can help identify the types of processing operations required to accomplish the purpose, whereas the nature of these processing operations, in turn, may allow the controller to further specify and delineate purposes.

Beyond compliance with the concrete documentation obligations under Article 30(1) of the GDPR, maintaining a detailed overview of the purposes and their associated processing operations is also a necessity in the context of assigning GDPR roles in complex data processing environments, where personal data are processed by multiple parties across longer processing chains, potentially for multiple, distinct purposes. The GDPR mandates that the parties involved in processing chains contractually agree on their respective roles under the Regulation (eg the roles of controllers and processors, but also processor and sub-processor), alongside their respective GDPR compliance obligations. The determination of the roles must take place in a manner that objectively reflects the nature and the extent of the parties’ involvement in data processing.^26^ Having a holistic understanding of the processing operations across the chains of processing, and their exact purpose(s), is therefore essential for a correct allocation of the GDPR roles, as well as for contractual clarity among the parties in relation to their respective obligations and potential liabilities.^27^ As such, purpose specification, by virtue of its inextricable link to the design of processing operations, is also vitally important for ensuring the collective GDPR compliance by multiple parties involved in complex processing environments.

Based on these considerations, it is clear that purpose specification plays a key role in controllers’ overall GDPR compliance obligations. The next section explores the question of how purposes should be specified under the GDPR.

## III. HOW NARROWLY OR BROADLY SHOULD PURPOSES BE SPECIFIED?

Considering the critical role of purpose specification for key compliance elements, it is surprising that neither the GDPR nor the interpretive guidance of it by the EDPB prescribes how purposes should be specified. This is largely due to the fact that the term ‘purpose’ is not explicitly defined under the GDPR.^28^ On the one hand, the concept of ‘purpose’ in the sense of the GDPR is somewhat intuitive: put simply, ‘the purpose pertains to the aims of processing’, as also stated by the EDPB.^29^ On the other hand, the lack of a more precise legal definition makes it challenging for controllers to specify the purposes for which they intend to process the data in a manner that is demonstrably compliant with the GDPR.

The lack of more prescriptive guidance by the EDPB in relation to purpose specification reflects the approach adopted by the EDPB’s predecessor Article 29 Working Party (hereafter WP29), in the context of purpose specification under the previous DPD. In their Opinion 03/2013, WP29 states the following:

Vague or general purposes such as ‘improving users’ ‘experience’, ‘marketing’, ‘IT-security’ or ‘future research’ will - without more detail - usually not meet the criteria of being ‘specific’. However, the degree of detail in which a purpose should be specified depends on the particular context in which the data are collected and the personal data involved.^30^

This lack of precise guidance on purpose specification by regulatory authorities is at odds with the controllers’ need to define their purposes of data processing in a sufficiently specific manner that enables demonstrable compliance with the GDPR. This unmet need of controllers is reflected in the growing body of literature exploring various approaches to establishing consistent frameworks for purpose specification.^31^ Recently, in certain domains of data processing traditionally characterized by a high reliance on standardized and interoperable information technology systems, community-wide shared standards and best practices have emerged around purpose specification. For example, Fouad and colleagues have proposed a taxonomy describing purposes of internet cookies in a standardized manner (also known as ‘purpose standardization’), with the aim of enabling GDPR compliance.^32^ However, many other areas where personal data are processed lack a similar level of technical interoperability and standardization, making it more challenging to develop GDPR-compliant comprehensive taxonomies of purposes for the processing of personal data. This is, for example, the case with scientific research. The concrete challenges and their potential solutions associated with purpose specification in scientific research will be discussed more extensively in the next section.

Owing to the difficulties with developing comprehensive taxonomies of purposes, it is useful to approach the question of purpose specification from a conceptual standpoint. Below, we outline a proposed general framework for specifying purposes for processing personal data in a GDPR-compliant manner. Our proposed framework consists of rules for defining a valid purpose under the GDPR, which have been derived from the data protection principles in Article 5(1) of the GDPR, their implementation through subsequent GDPR articles, as well as other concrete GDPR-based obligations of the controllers.

From the GDPR, the following key preconditions for specifying a valid purpose can be distilled:

– Purposes of the processing must be specific^33^ and specified.^34^
– A purpose must be specific enough to inform the processing, ie it must be possible to demonstrate that processing operations are necessary for the stated purpose.^35^ The assumption that the purpose must reflect the associated processing is supported by cumulative requirements for a valid consent under the GDPR: Article 6(1)(a) of the GDPR states that consent must be provided by the data subject for a specific purpose, while according to Article 7(1) of the GDPR, the controller must be able to demonstrate that the data subject has consented to the processing of their data. Furthermore, it must be possible to assess whether data are accurate and up to date with regard to the purpose for which they are processed.^36^
– A purpose must allow the determination of how long the data should be processed.^37^ This implies that a valid purpose cannot give rise to an indefinite duration of processing without a clearly determined or determinable end-result. Such understanding of a purpose is also reflected in the French translation of the GDPR, which uses the word ‘finalité’ in reference to purpose: finalité, similar to the English ‘end’ as in ‘means to an end’, implies that the activity in question has a clear endpoint marking its accomplishment or conclusion. If establishing the precise duration of processing is not feasible, at a minimum, clear criteria for determining whether the purpose has been achieved must be articulated.
– The controller must be able to demonstrate that the envisaged data that are collected are necessary for achieving the purpose.^38^ Of note, a valid purpose does not have to explicitly specify the data subjects or categories of data subjects, data types, and recipients or categories of recipients. This interpretation can be derived from Article 4(7) of the GDPR, which states that: ‘the controller […] determines the purposes *and* means of the processing of personal data’ (emphasis added). The wording of Article 4(7) of the GDPR implies that purposes do not have to be specified narrowly enough so as to directly encompass all the means of the processing (eg categories of data subjects, types of the data, categories of recipients). Rather, the controller has the discretion to choose the appropriate means for achieving the specified purposes, though these must be specified before commencing the processing.^39^ As a practical example, ‘determination of a suitable biomarker to predict the progression of disease XY’ would qualify as a purpose. It leaves the discretion to researchers to investigate various data types that might qualify for such biomarker without having to know upfront which data types may be most suited. The information provided to data subjects must specify which data types will be investigated, but this can be done outside the actual purpose definition. A purpose can therefore be phrased without a prior hypothesis about which datatypes will eventually constitute the biomarker.

To be able to demonstrate compliance with the principles of the GDPR as required under the newly introduced accountability principle of Article 5(2), purpose(s) of processing personal data must be specified in a manner that fulfills *all* of the conditions listed above. Hence, a valid purpose under the GDPR must be specific enough to inform the design of the processing, ie determine what processing is or is not included, to justify collected data types and chosen category of data subjects, and should aim at an achievement that allows to determine the duration of the processing for this purpose. This compliance-enabling approach to purpose specification is also reflected by the EDPB in their Guidelines 2/2019: ‘The purpose of the collection must be clearly and specifically identified: it must be detailed enough to determine what kind of processing is and is not included within the specified purpose, and to allow that compliance with the law can be assessed and data protection safeguards applied.’^40^

Correctly defining the purpose for the processing is not always straightforward in a more complex processing chain and may necessitate multiple steps of refinement, to be performed in an iterative manner. Namely, the controller may need to first identify preliminary (‘draft’) purpose(s), and subsequently refine it/them in a manner that complies with the GDPR requirements for purpose specification. To help controllers with this task, we recommend that controllers adopt an iterative approach to purpose specification by following the steps described below:

*Step 1. Define the aim of the processing and its endpoint as a first ‘draft purpose’* : what is the reason for the collection and/or otherwise processing the personal data? What result is to be achieved? The formulation of the draft purpose should be such that it entails a defined or definable endpoint that is to be achieved, which will mark the conclusion of data processing for that purpose. An open-ended objective such as ‘cancer research’, for example, does not enable the controller to define concrete endpoint(s) corresponding to the accomplishment of the purpose.

*Step 2. Ascertain the adequacy of purpose specification:* is the (draft) purpose specific enough to inform the design of the processing required for achieving the purpose? What are the envisaged categories of data subjects concerned and the data types to be collected (or otherwise processed)? Can the selection be justified by the purpose? (Phrased another way, why *these* categories of data subjects and data types, and not others?) Under the GDPR, a valid purpose must be specified in a manner that enables the controller to justify the design of the project by demonstrating the necessity of the chosen essential means (including categories of data subjects and data types) in achieving the purpose. Where the formulation of the draft purpose lacks this information, the controller will need to incorporate a more detailed description of the envisaged data processing activities. This means that a purpose specification will often need to include more details than the mere result that is to be achieved. The ‘how’ and the ‘why’ will become part of the description to elucidate the choices made. A properly defined purpose promotes accountability for data minimization: if the stated purpose can be achieved without certain data, then those data should not be collected in the first place.

*Step 3. Assess whether further processing of personal data takes place:* can the controller clearly justify that each processing of personal data is necessary to achieve the defined purpose? Building on the description of intended processing performed in Step 2, the controller should map out the envisaged data processing operations in a reasonably comprehensive manner, reflecting the totality of the information available to the controller regarding the planned processing. Subsequently, the controller needs to evaluate whether a particular processing operation is strictly necessary to achieve the purpose for which the controller seeks to obtain the data. If the result could be obtained without a certain processing operation, the processing operation in question constitutes further processing of the data for another purpose that the controller must also specify in a GDPR-compliant manner.

*Step 4. (In complex processing environments), ascertain that no unnecessary purpose* *fragmentation takes place:* along a processing chain involving a number of steps, are the different processing operations performed for multiple distinct purposes, or for a single overarching purpose? To assess whether purposes are distinct or part of an overarching purpose, the controller should determine which of the defined purposes would by itself be a sufficient result that justified data collection and where subsequent processing operations and purposes are opportunistic rather than necessary. In some complex processing environments, there is a risk that the controller could misattribute different processing operations to distinct purposes, while in reality they are performed to achieve a single overarching (macro) purpose. This could happen in particular where the processing along a processing chain uses different subsets of the data. Nevertheless, these different phases may be necessary to achieve only interim results, which alone would not justify the collection of data. An example could be the establishment of a database or a research cohort, which constitutes a clearly defined, achievable endpoint, but in itself is not a sufficient purpose to justify the processing. It is only in view of the intended subsequent use of the data stored in the database that the collection of the data can be justified and the corresponding processing designed (we draw the reader’s attention to the section below on ‘Micro-level and macro-level purposes in research’ for a more detailed analysis of this scenario based on a real-world example).

With respect to Step 4, it is essential that controllers adopt a careful and differentiated approach to purpose specification, that is, by taking into account different phases of processing throughout the data lifecycle. In this respect, the EDPB cautions in its Guidelines 07/2020^41^ that in complex processing chains involving several actors, different processing operations along the chain may appear disconnected (and hence, performed for independent purposes) on a micro-level, whereas a closer inspection of the influence and definition of means might reveal that the processing operations are pursued to achieve the same macro-level purpose that provides a justification for the overall collection. We would like to add that, even where only one controller is involved, the separation of the primary purpose and further processing is not always clear.^42^ The above questions are designed to guide the controller in this separation.

In the following section, we apply the proposed iterative approach to purpose specification to the context of scientific research, illustrating how the approach can help researchers identify and refine the purpose(s) for processing personal data under different relevant scenarios.

## IV. DEFINING VALID PURPOSES FOR SCIENTIFIC RESEARCH

Within the legal framework established by the GDPR, ‘scientific research’ is an important concept whose exact meaning can differ across contexts. Recital 159 of the GDPR frames the concept in a broad manner:

[...] For the purposes of this Regulation, the processing of personal data for scientific research purposes should be interpreted in a broad manner including for example technological development and demonstration, fundamental research, applied research and privately funded research. [...]^43^

However, depending on the context, ‘scientific research’ can also have a narrower meaning, referring to a particular research project pursued by the controller. This is reflected in the EDPB Guidelines 05/2020, which define scientific research as ‘a research project set up in accordance with relevant sector-related methodological and ethical standards, in conformity with good practice’.^44^

Given this semantic ambiguity, below we discuss multiple scenarios of processing personal data for scientific research, in a broad sense of the term. Collectively, these scenarios capture the following cases: (i) the researcher processes personal data to perform a research project according to the design that the researcher developed; (ii) the researcher shares the data with another researcher for the latter’s research project; (iii) the researcher deposits personal data in a dedicated data repository in order to make the data widely available for qualified third parties pursuing their own research projects.^45^

### IV.A. Pursuit of an Own Research Project

As discussed previously, the controller must define the purpose(s) of processing in a manner that is sufficiently specific to inform the design of the processing. Translated to the research context, this means a valid purpose under the GDPR must allow the design of the data processing activities envisaged within the intended research project. The controller must also be able to specify the personal data categories and the categories of data subjects to be included in the proposed research activities. In practice, this can be accomplished by elaborating an appropriate data analysis methodology and a comprehensive data analysis plan prior to initiating the data collection, with a clear link between the purposes of the study and the description of the processing. It follows that a GDPR-compliant purpose in scientific research should be formulated such that its scope is focused on an individual research project. The iterative approach to purpose specification outlined in the previous section helps explain the logic behind this conclusion.

As per the proposed framework for purpose specification, a valid purpose must be achievable and have a clearly defined endpoint. That is, it must be able to answer the question, ‘what result is to be achieved?’ A broadly formulated purpose, such as ‘research on new therapeutic approaches in oncology’, will not fulfill this requirement. Another insufficiently specified (and hence, invalid under the GDPR) formulation of the purpose would be ‘finding new therapies in oncology’: although this formulation describes the nature of the achievement sought by the controller, it fails to provide an affirmative answer to another key question, namely, ‘is the (draft) purpose specific enough to inform the design of the processing required for achieving the purpose?’ In order to determine which processing activities are (not) included to achieve the purpose, the controller must further specify the purpose. Effectively, in this scenario, a correctly formulated purpose should directly encapsulate the specific research question(s) that have motivated the design of the planned research project. When specified in this manner, the purpose will also inform a research methodology, which justifies the (essential) means of processing, eg the categories of data subjects concerned (corresponding to study recruitment or inclusion criteria) and the personal data categories processed (corresponding to elements of the data analysis plan). The outcome of the intended processing, and hence the end-result marking the achievement of the purpose, will be the generation of knowledge based on the question of the research project.

Specifying the purpose in a manner that not only informs the design of processing but also links it to a well-defined end-goal may appear too narrow and hence overly restrictive for the pursuit of research. However, this interpretation is in line with the broadly accepted requirements for good scientific research practices. For example, the European Science Foundation (ESF), in its ‘Good Scientific Practice in Research and Scholarship’ policy briefing, lays down the same substantive requirements:

All research should be designed so that it has a clear objective, either answering a valid scientific question or, in scholarship […]. The design of the study must be robust, the procedures proposed technically feasible and the intended methods of analysis appropriate. Protocols and plans should, therefore, be written in clear and unambiguous terms. They should include specific details of the aim, materials, methods, time schedules and analytical approaches to be used.^46^

As such, the GDPR-derived criteria for purpose specification in the context of pursuing a scientific research project are closely aligned with the requirements dictated by good scientific research practices. Hence, by following the latter, researchers will also effectively lay the groundwork for specifying purposes of processing personal data in a GDPR-compliant manner.

Nevertheless, a difference is to be made between a purpose definition that reflects the scientific protocol and the narrative that is to be provided to the data subjects. The latter needs to be done in accordance with Article 12 GDPR and should focus on relating the scope and the consequences of the processing.

It is debatable to what extent it will always be possible to specify the full purpose prior to the data collection. Recital 33 GDPR, focusing on scientific research, states that this will not always be feasible. An example can be found in population-level cohorts of a longitudinal character where the precise research questions emerge over time and are determined also by the disease trajectories observed among the research participants. Purpose specification in such cases would have to be more generic based on the range of research questions that are to be pursued with an emphasis on how the research questions will be determined and with the possibility to inform data subjects more precisely once the individual research questions crystallize. This is typically done through the webpage of such cohorts as well as in regular communication (eg via newsletters) provided to the participants of these cohorts.

### IV.B. Data Sharing with an External Researcher Pursuing their Own Research

An example of such a scenario may arise where a research organization that has established a cohort (in the following also called the ‘initial controller’) is approached by an external researcher with a request to access and use the data, for a study the external researcher has designed and is pursuing independently. (Re)use of the data by the external researcher therefore gives rise to a *new* purpose of processing under the GDPR. The initial controller will assess such requests individually and decide whether to disclose data to the requestor. Reasons for a decision to disclose the data may include, for example, seeking greater visibility for the initial controller’s cohort, or shared research interests with the external researcher, which feeds into an intention to pursue collaborative projects in the future. The initial controller may also be motivated by a wish to further scientific advances or to enable reproducibility studies. With respect to the recipient researcher’s purposes when requesting data access: the original research project for which the initial controller collected the data was designed prior to the intended recipient’s request. Hence, in order to comply with the GDPR, it is necessary to specify a new purpose, for which the recipient researcher’s organization will be the controller. This effectively precludes joint controllership, making the party that initially collected the data for its purposes and the intended recipient independent controllers for data sharing and data use for own research, respectively.^47^ The initial controller may disclose the data to allow the external researcher to pursue the validation, but they will not be involved any further. The processing of the initial controller is limited to the disclosure, without exercising influence on the research by the recipient, or any further processing following the disclosure. This has to be reflected in the purpose specification.

Importantly, ‘data sharing’ or ‘data sharing for research’, while describing the overall nature of the processing, does not provide the justification of categories of data subjects and personal data categories undergoing processing. These essential means depend on the objectives and the research question of the external researcher requesting data access. Data sharing is a processing operation rather than an achievement. Only by specifying that sharing takes place for the external party’s research project is it possible for the initial controller to design processing in a manner that enables compliance with the GDPR principles. For example, by ensuring that the shared data are limited to the data strictly necessary for addressing the external party’s research question, the initial controller could demonstrate its compliance with the data minimization principle. In view of these considerations, it is suggested that a valid purpose, in the sense of the GDPR, should be framed along the lines of ‘data contribution to the external party’s research project’, where the specific research project of the external party provides the justification for the scope and nature of processing, including with regard to personal data categories and categories of data subjects.

Data sharing by the initial controller could also take place for a defined scope of research by the intended recipient beyond a specific project as long as the data use conditions agreed upon previously allow this. An illustrative example in this respect is generation of large-scale genomic data, such as whole-exome or whole-genome sequence (WES/WGS) data. WES/WGS data constitute valuable resources for exploratory research, particularly where they are associated with other information (eg health data and family medical history) from a well-defined clinical or research cohort. Researchers may generate WES/WGS data from biosamples acquired together with relevant clinical information. These data can be utilized by researchers iteratively, whereby results of initial data analyses inform the refinement of the research question, potentially leading to the formulation of new related hypotheses based on recently generated insights.^48^ The investment into WES/WGS data is justified by their high utility for exploratory research, which, however, depends on the accompanying clinical data. Contractually limiting external researchers’ use of the clinical data obtained to one-off applications such as performing single, narrowly defined data analysis, would result in undue administrative burden for the subsequent use of the WES/WGS data generated by the external researcher. Namely, the external researcher would be required to obtain a new approval from the initial controller each time it seeks to perform a new query on the WES/WGS data and linked health data in the context of evolving research question(s).

A more research-enabling approach would be for the initial controller to contractually permit the use of its data by the specified recipient for an entire area of research. Here, the initial controller has the purpose of contributing data to the defined research areas of the recipient researcher, provided that doing so by the initial controller is in line with the data use conditions defined vis-à-vis the data subjects. Using our proposed 4-step approach to purpose specification, it can be shown that data contribution by the initial controller for the recipient controller’s use within defined research areas constitutes a valid purpose under the GDPR. This purpose allows the initial controller to answer all of the required questions; for example, the initial controller can specify the categories of personal data being shared, alongside the categories of the data subjects concerned, owing to the fact that the transfer is taking place for a defined area of research. The initial controller can also describe what processing is and is not included as part of data sharing, while the achievement of the purpose pursued by the initial controller can be defined as successfully disclosing the data to the recipient. It is the decision of the initial controller to choose between the specific purpose of sharing data for an individual project only or for defined categories of purposes. This will lead to different specific purposes on the side of the initial controller: contribution of data toward a single research question or a range of research questions.

However, it is worth highlighting that the aforementioned purpose of contributing data toward a range of research questions is only valid (i) from the initial controller’s standpoint, and (ii) with respect to the contribution of the data to a particular recipient. The possibility to cover areas of research, ie a range of research questions, does not stretch to the subsequent use of the data by the recipient controller for its research project(s). The research pursued by the recipient will have to comply with the requirements of purpose specification, requiring the recipient to carry out the 4-step analysis from its own perspective. The areas of research for which the initial controller contributes the data (which can be sufficient to specify the initial controller’s purpose for data contribution) are not a specific enough purpose for the recipient controller to describe its own research processing. Instead, each distinct research question for which the data are processed by the recipient will constitute a separate purpose under the GDPR. Similarly, from the initial controller’s point of view, although the disclosure of the data to the recipient for a defined area of research constitutes a valid GDPR purpose, this purpose only covers a particular recipient controller. Data sharing by the initial controller with another recipient should be framed as an activity carried out to accomplish a different purpose within the meaning of the GDPR.

The practical consequences of these considerations are reflected in the information obligations. Where an initial controller shares data with a recipient for the recipient’s defined research project, the initial controller needs to inform the data subject about the specific recipient and the specific research project, being able to cover the information obligations of the recipient under Article 14 GDPR, as well if the recipient provides the relevant information to the initial controller. On the other hand, where the initial controller decides to share data for an area of research pursued by the recipient, it would also inform the data subject, but in this case about the range of research questions that will be pursued by the recipient. By contrast, informing the data subject about each research question, to the extent applicable under GDPR Article 14(5)(b), becomes the recipient controller’s obligation (as the information provided by the initial controller on the areas of research will not suffice).

An initial controller may also decide to make data available to a range of (independent) recipients for areas of research. However, this scenario is different, being akin to the establishment of a data repository as a first step prior to subsequent instances of data disclosure to recipient users. Corresponding scenarios are addressed in the next section.

### IV.C. Contributing Data to Biobanks and Data Repositories

The scenario above describes an incidental sharing of data that are held by the initial controller for its own research purposes. The initial controller may also decide to make data systematically available to the research community for defined areas of research, as opposed to assessing the acceptability of data sharing only after a data access request has been made. Such systematic availability is typically facilitated through data repositories or, where data are held in conjunction with biosamples, a biobank.^49^

There are different processing steps along the data lifecycle in repositories, which are in large part determined by the choice of the repository. Some repositories only accept data in certain formats, which may require transformation of the data into specific semantic data models. Once included in the repository, data can be discovered by researchers from data catalogues and access can be requested for a defined research project’s purpose. Personal data may be processed to generate the information necessary to be included in the data catalog of the repository that make the data findable, such as maximum and minimum values within the dataset, which can be established by consulting the underlying dataset. The data access decision can follow different approaches. In some cases, the access decisions are taken by the data access committee of the initial controller that submitted the data to the repository; an example of this access modality is the European Genome Phenome Archive (EGA) hosted by the research infrastructure European Bioinformatics Institute (EBI) and the ELIXIR Node in Spain.^50^ In other cases, the decision is taken by the central data access committee established through the repository’s host, where such decision can be taken independently of the initial controller; an example is the database of Genotypes and Phenotypes (dbGaP) operated by the National Center for Biotechnology Information (NCBI) in the USA.^51^ Following a decision to grant access, data are disclosed to the requesting scientist, who can either download the data or process them in a dedicated secure processing environment, depending on the data access conditions of the repository.

In the processing chain, the pursuit of a specific research project by the external researcher provides the basis to specify purpose(s) of processing in a GDPR-compliant manner. This is the responsibility of the data-requesting researcher, whose organization acts as a controller under the GDPR with respect to the processing following data disclosure. On the other hand, the step of disclosing the data requires processing operations where the controller is either the initial controller or the repository, depending on the precise functional roles of the parties. For these processing operations, the purpose of the controller should be contribution of data to the external researcher’s specific research project, which is identical to the scenario discussed above.

In the research context, the reason for a deposition of data in a repository is often the initial controller’s aim to make data available for future research, eg based on institutional policies or requirements of public funders.^52^ The initial controller’s purpose, in the sense of the GDPR, should be defined accordingly, ie in relation to making data available through the repository with a view to downstream sharing with external data users. An important element here as compared with our example in the previous section is an added step preceding data sharing with downstream recipients. First, rules for data availability are established, which includes not only the categories of recipients and the areas of research for which data can be disclosed, but also the mechanisms for taking decisions regarding data disclosure. Under these circumstances, making data available constitutes a valid purpose in the sense of the GDPR, not only for its specificity, but also for having a well-defined end-result. This purpose of creating availability is achieved once the data are made findable through the catalog of the repository and can be requested by external researchers.^53^ All the processing steps in the processing chain that take place before the data are made findable will therefore be necessary to achieve the purpose. This purpose does not cover the actual downstream sharing for a specific research project, which will require the specification of another valid purpose.

When pursuing the purpose of making data widely available through a repository, the exact nature of the processing operations required to achieve the purpose will depend on the choice of the repository. While some repositories ask for only very minimal data description for their catalogues and require no data curation (eg EGA), others have more elaborate requirements (eg dbGaP) that could even warrant change of controllership for the downstream data disclosures. Depending on the choice of repository, the deposition in the repository may also include a transfer to a third country (eg dbGaP) or international organization (eg EGA), as well as subjecting the data to different access and use conditions for future research reuse. This implies that the choice of repository will generally amount to more than deciding on the means of processing to achieve the purpose specified by the initial controller. Rather, the choice of the repository will typically influence the nature of the processing in a substantive manner, thus bearing on purpose specification.^54^

To specify the purpose in a GDPR-compliant manner, we once again turn to the guiding question: ‘is the (draft) purpose specific enough to inform the design of the processing required for achieving the purpose?’ It follows that the specified purpose for depositing data in a repository for future research use must include the choice of repository or otherwise provide information about the related processing operations. As with other scenarios of data sharing, the areas of research or, if applicable, other relevant purposes (eg policy development) for which the data are to be made available should be captured in the initial controller’s purpose. The availability of data for a scope of purposes for which data can be lawfully disclosed to third parties and according to defined procedures is a legal status that data assume through the series of processing operations leading up to the inclusion of the data in the repository.

To cover these operations, the decision-making regarding future reuse and therefore potential changes in controllership needs to be captured, as applicable. We therefore recommend that the purpose for a deposition in a repository for future research should be framed as ‘making data available for future research in the area of NN through the repository XY’. NN in this case includes the data use conditions. For fairness and transparency reasons, the implications of the choice of repository, such as the data governance framework, potential change of controllership, and transfers to a third country should be explicitly included in the description of the purpose. In particular, where consent is supposed to be the legal basis under the GDPR for making data available for future research, this comprehensive information is essential to obtain a valid consent.

This requirement can be seen as limiting in that it necessitates that the initial controller actively restrict its choice of repositories through which the data can be made available. However, it is worth noting that the GDPR-derived requirement to specify the purposes of processing in this manner is in line with the spirit of existing bioethics guidance concerning valid informed consent in biobanking research. For example, according to the *International Ethical Guidelines for Health-related Research Involving Humans* by the Council for International Organizations of Medical Sciences (CIOMS) in collaboration with the World Health Organization (WHO), a valid informed consent to future use of biosamples in biobanks and/or data in data repositories should specify:

the purpose^55^ of the biobank, the conditions and duration of storage; the rules of access to the biobank; the ways in which the donor can contact the biobank custodian and can remain informed about future use; the foreseeable uses of the materials, whether limited to an already fully defined study or extending to a number of wholly or partially undefined studies; the intended goal of such use, whether only for research, basic or applied, or also for commercial purposes, [...].^56^

## V. MICRO-LEVEL AND MACRO-LEVEL PURPOSES IN RESEARCH

The EDPB introduces in its Guidelines 07/2020 the notion of micro-level and macro-level purposes to help controllers correctly define purposes of data processing under complex processing chains. Micro-level purposes are pursued during specific stages of processing along a processing chain, whereas the macro-level purpose describes the overall intended achievement justifying the processing. Micro-level purposes are particularly relevant in research. Recital 33 of the GDPR states that it is often not possible to fully identify the purpose of personal data processing for scientific research purposes at the time of data collection.^57^ This means that only micro-level purposes can be specified, whereas the macro-level purposes can only be described on an overall generic level, referred to as areas of research, at least at the time of the data collection.

Population-level studies such as birth cohorts are a typical example of purposes that cannot be fully identified at the time of collection: the precise research question will depend on the health and disease progression of the research participants, which can only be defined at a later stage. Revisiting Step 4 in the purpose specification methodology described above, it becomes clear that building up a cohort without pursuing any research questions would not be justified. As such, the establishment of the cohort *per se* is not an independent purpose, even though the establishment of the baseline of the cohort often leads already to a first publication; the cohort is designed based on an envisaged scope of research questions to be addressed later. Therefore, the establishment of a cohort merely constitutes accomplishment of a micro-level purpose across the processing chain whose macro-level purposes will be further specified once downstream research questions are clearly defined.

A recent example of a controller that misjudged a micro-level purpose as an independent purpose can be found in the June 30, 2022 Opinion, pursuant to Article 110 of the Italian Personal Data Protection Code (Legislative Decree June 30, 2003) and Article 36 GDPR, by the Italian data protection supervisory authority, the Garante per la Protezione dei dati Personali (hereafter, Garante).^58^ In the case addressed by the Opinion, a university hospital had defined separate purposes for the following activities: (i) establishment of a database on which to build future analyses and studies aimed at improving knowledge and clinical practice in the field of pathologies of the thorax; and (ii) the specific studies to be performed in these areas as well as the further studies beyond the specified scope of questions. The Garante emphasized in its Opinion that the collection (generation) and storage of data in the database without linking the processing to the subsequent research in the defined areas would not have been legally possible. The studies in the envisaged areas are therefore the macro-level purpose for which the database is built. The creation of a database by itself is not a valid purpose; it is no ‘end’ in itself, but a continued processing to answer downstream questions envisaged for the creation and design of the database, and which become progressively more concrete.

## VI. RELEVANCE OF PURPOSE SPECIFICATION FOR THE DETERMINATION OF THE LEGAL BASIS

Following the discussion of purpose specification under different scenarios relevant to scientific research, in this section we elaborate on how purpose specification can help researchers ensure that their organizations are compliant with their GDPR obligations as controllers. We use the example of compliance with the principle of lawfulness, which, among other things, obliges the controller to select a basis to legitimize processing of personal data from the six options commonly referred to as GDPR legal bases listed under Article 6 (1) of the GDPR. When the controller processes special categories of personal data, which include health data and genetic data, the controller must additionally select a suitable Article 9(2) exemption from the 10 available options.

We choose to focus on this particular aspect of GDPR compliance because the absence of an appropriate legal basis to legitimize processing has been reported as the single most common GDPR violation cited in fines imposed by data protection authorities. For example, a 2022 quantitative analysis of all fines issued by national data protection authorities revealed that processing personal data without a sufficient legal basis had been cited in 409 cases out of the total 856 decisions (48 per cent), significantly exceeding the proportion of other cited GDPR violations that resulted in a fine.^59^ Another reason for focusing our subsequent analyses on legal basis is the conceptually challenging nature of interpreting Article 6(1) and 9(2) GDPR requirements in the context of secondary data use in scientific research, including a widespread confusion around whether identifying a valid legal basis is even necessary.^60^ As the analysis below will demonstrate, a GDPR compliance approach with purpose specification at its core allows to not only ascertain whether a legal basis is required, but also determine which of the available legal bases are appropriate for the data processing at hand.

As highlighted previously, the EDPB states that the appropriate legal basis must be clearly connected to the specific purpose of processing. When processing personal data for research purposes, the following Article 6(1) legal bases are of particular relevance:

– The data subject’s consent to one or more specific purposes (Article 6(1)(a));
– Legal obligation (Article 6(1)(c));
– Task carried out in the public interest (Article 6(1)(e)); and
– The legitimate interest pursued by the controller or a third party (Article 6(1)(f)).

Of the four legal bases, Article 6(1)(a) makes the most direct reference to purpose specification, reiterating that the validity of consent as the legal basis for processing personal data depends on whether the consent has been obtained for one or more specific purposes. The connection between Article 6(1)(c) of the GDPR and the concept of purpose is more indirect. Article 6(1)(c) as a legal basis to process personal data requires the existence of a legislative act mandating the controller to undertake the relevant processing activity. Article 6(1)(c) is complemented by Article 6(3) of the GDPR, which states that in this case the law must also determine the purpose of the processing, hence linking the legal basis with the purpose also for Article 6(1)(c) GDPR. A direct purpose reference is again included in Article 6(1)(f) of the GDPR, albeit summarily: the processing must contribute to purposes in the legitimate interest of the controller or a third party. It is this legal basis that makes a clear distinction among the overall aim that motivated the processing, the interest of the controller, and the more granular aims, the purposes, that inform the design of the processing and that are focused exclusively on the results of that processing. The distinction between ‘interest’ and ‘purpose’ is an important one in the EU’s data protection legislative framework. WP29 clarified this distinction in their Opinion 06/2014:

The concept of ‘interest’ is closely related to, but distinct from, the concept of ‘purpose’ [...]. In data protection discourse, ‘purpose’ is the specific reason why the data are processed: the aim or intention of the data processing. An interest, on the other hand, is the broader stake that a controller may have in the processing, or the benefit that the controller derives - or that society might derive - from the processing.^61^

There is an overlap in scope between Articles 6(1)(f) and 6(1)(e): the latter also refers to an interest; the relevant interest here is that of society. In the interest of society, tasks are assigned to a controller, or are carried out under the official authority vested in a controller. The respective interest has to be defined by the legislator: a legal measure is needed to invoke Article 6(1)(e).^62^ A difference between the legal bases is that Article 6(1)(e) refers to a *task* rather than to purposes. Here, greater flexibility is provided. A task in the public interest can be framed in a broad way where it is established through a mandate or mission vested in a public or private body by national or EU law.^63^ On the other hand, a task can also be relatively narrow where processing is only applicable to a party’s supporting role in a wider context of processing. An example of a narrowly defined task is the assignment of the responsibility for technical and organizational measures to ensure a level of security appropriate to the risk to a processor, which, among others, include encryption and pseudonymization of data. Purposes in the context of the tasks assigned based on Article 6(1)(e) therefore must fall under the scope of the assigned tasks. The controller needs to perform a test to demonstrate that the purposes envisaged are covered by the mandate, mission, or task given to the controller.

## VII. UTILITY OF PURPOSE SPECIFICATION IN IDENTIFYING A SUITABLE LEGAL BASIS FOR DATA PROCESSING IN THE RESEARCH CONTEXT

In what follows, we demonstrate, based on relevant examples in the context of research, how purpose specification can help researchers identify a suitable legal basis for data processing.

### VII.A. Publication of Research Results

Publication of research results follows the finalization of a defined research project. Since the publication is a step taking place after the research questions have been answered, it may appear to necessitate a separate purpose under the GDPR, in particular as it requires considerable related processing. For example, such related processing operations may include transforming part of the raw data into a more intuitive or presentable medium (eg tables or figures) and providing access to the raw data, possibly in its entirety, for reviewers as part of the standard scientific peer-review process. To assess the relevance of the processing necessary for the publication of research results, it is instructive to revisit the proposed definitions of scientific research under the EU’s data protection legal framework.

As discussed previously, according to the EDPB Guidelines 05/2020, (paragraph 153),^64^ scientific research in the context of the GDPR means a research project set up in accordance with relevant sector related methodological and ethical standards, in conformity with good practice. We use as reference for good practice in scientific research the ‘Good Scientific Practice in Research and Scholarship’ policy briefing of the ESF, and for methodological and ethical standards in the sector of health-related research, the CIOMS *International Ethical Guidelines for Health-related Research Involving Humans.*

The ESF defines research in rule 1:

Scientific research and scholarship are diverse and multifaceted activities embracing a wide range of intellectual and practical endeavours. These include theoretical studies, experimental work and surveys, as well as the verification, further analysis and extension of earlier work. The objective is always to extend human knowledge and our understanding of the physical, biological and social worlds.^65^

It further elaborates in rule 38: ‘Publication in a peer-reviewed journal or as a scholarly book is an important stage in the scientific process, marking the point when data, theories, interpretations and paradigms formally enter the public domain.’^66^

CIOMS also states in Guideline 24 the following:

Public accountability is necessary for realizing the social and scientific value of health related research. [...] Researchers have a duty to make the results of their health-related research involving human beings publicly available and are accountable for the completeness and accuracy of their reports.^67^

Based on this information, we can proceed to specify the purpose in a GDPR-compliant manner by revisiting Step 4 of the proposed approach to purpose specification, which concerns complex chains of data processing consisting of multiple phases of processing. Namely, we can examine whether a particular achievement along the processing chain, in and of itself, would be necessary and sufficient to justify the data collection, absent any downstream processing. Translated to the case of publishing scientific results, this essentially means asking the following question: can the data collection and the conduct of research be justified if results are not to be published? Given the widely accepted ethical and methodological standards governing scientific research, it is clear that the answer to this question is ‘no’. Therefore, we suggest that the publication of research results, including the archiving of related data for reproducibility reasons as required for the integrity of scientific research, should be considered as part of the primary purpose of knowledge generation on the defined research question and is therefore covered by the same legal basis. In other words, no independent legal basis needs to be established; the legal basis applicable to the research project also applies to the publication of the project results. This is particularly relevant where consent is the legal basis for the research: no separate consent is needed for pursuing the research and the data processing for the publishing of results.^68^

### VII.B. Pseudonymization and Anonymization of Data

Pseudonymization or, where possible, anonymization of personal data, is an integral element of processing carried out routinely as part of research projects. It may therefore appear that pseudonymization is a necessary processing step for research and hence covered by the GDPR legal basis under which the research is pursued. However, posing the question, ‘can the controller clearly justify that each processing of personal data is necessary to achieve the defined purpose?’ (Step 3 of our proposed approach to purpose specification), reveals that the necessity for pseudonymization or anonymization does not arise from the research question: pseudonymization or anonymization does not advance the pursuit of a specific research project and may even inhibit the researcher’s objectives by introducing additional hurdles to the use of the data. Hence, it is helpful to take a step back and ask the starting question from the proposed purpose specification framework: ‘what is the reason for the processing?’ In doing so, it becomes clear that pseudonymization or anonymization is necessary to comply with the obligations laid down in Article 89(1) of the GDPR for scientific research. Consequently, the legal basis for the processing is rather Article 6(1)(c), ie a legal obligation based on Article 89(1). In some cases, where these obligations have become part of mandatory safeguards under Article 9(2)(j) or Article 9(4) of the GDPR for processing health and genetic data in the context of scientific research, also national legislation may be relevant.

Anonymization may also take place instead of erasure of data at the end of a research project. Both anonymization and erasure are once again based on obligations under the GDPR, in this case, derived from the Article 5(1)(e) requirement (viz., the storage limitation principle) that data should not be kept in a form that permits identification of data subjects for longer than is necessary for the purposes for which the personal data are processed. Here, too, posing the initial question, ‘what is the reason for the processing?’, allows the controller to not only correctly specify the purpose of processing, but also to select the appropriate GDPR legal basis for the processing. The suitable legal basis remains Article 6(1)(c).

Anonymized data are no longer subject to the GDPR and can be used for any research, also beyond the scope originally agreed with the research participants as data subjects. This will be possible where data are anonymized as part of or following a research project. The situation is different where anonymized data are created as a separate dataset from the original pseudonymized data to enable research beyond the previously agreed scope. Here, the reason for the processing, ie anonymization, cannot build on Article 5(1)(e) because the original dataset remains in an identifiable format, ie no irreversible de-identification of the dataset has taken place.

Also, Article 89(1) is not applicable as the processing is not related to a legitimate research project, so the conditions of Article 89(1) are not fulfilled. The purpose for the processing is not required by law but driven by the research intentions of the controller: to generate an anonymous dataset that can be used for yet undefined research questions. Although some public bodies have received a legal mandate to create anonymous datasets that can be used for research purposes,^69^ other organizations will not be able to rely on such a mandate to anonymize data for future research. Consequently, a legal basis outside of an assigned task, mission or mandate, or a legal obligation must be established.

Consent is a possible option: the purpose to create non-personal data for future research questions that do not have to rely on identifiable natural persons allows a valid consent under the GDPR. The controller could also invoke legitimate interest as a legal basis under Article 6(1)(f) to have a wider pool of data flexibly available for research. To invoke a legitimate interest, however, a balancing test needs to be performed that weighs the interests of the controller against the interests of the data subject.^70^ To achieve a positive outcome of the balancing test, the reasonable expectations of the data subject must be taken into consideration. The data subject should be aware of the processing and should have an easy way to object. The information about the planned processing should therefore be made available at the time of collection or, at a minimum, prior to the anonymization, with the availability of an easy mechanism to object. It should be noted that without the transparency and possibility to object to the processing, the controller may not be able to rely on legitimate interest. The dedicated creation of a parallel anonymized dataset to rely on for future research outside the scope agreed with the data subjects may therefore not be possible without recontacting the data subjects.

### VII.C. Contributing Data to a Recipient Scientist’s Research Project

Above we described a scenario where the initial controller is approached to make their data available for an external scientist’s research project. We can now modify the scenario to one in which a project has progressed in a way that does not reasonably justify a collaboration with an external party, or the research may be outside the expertise of the data-holding researchers. In this case, no joint research will take place, precluding joint controllership under the GDPR. We have established that each such disclosure will require a separate purpose as the intended research purposes of the recipient scientist will be decisive for framing the purpose of the disclosure. This makes Article 6(1)(a) (consent) as a legal basis for the disclosure rather cumbersome, because each disclosure will require a separate consent.^71^

While the reuse of data in research is, in principle, in the public interest, eg by recognizing further processing for scientific research is seen as compatible under the GDPR, this is not sufficient to establish a legal basis.^72^ The mission of public research organizations and universities may be focused on their own in-house research, also highlighted by key performance indicators such as publications and patents. These tasks may not necessarily cover supporting the research of others (ie those who are not staff or students), depending on the precise scope of the legal mandate given to the research organization under the relevant national law. Consequently, purposes related to contributing data to research without own participation cannot always be justified by an own research mission. Article 6(1)(e), a task in the public interest, will therefore not always be a suitable legal basis because merely disclosing data without further engagement does not constitute research processing even though it is feeding into the research of another researcher.

An interesting alternative to investigate is legitimate interest. The purposes of legitimate interest must be related to a real and present interest.^73^ The EDPB clarifies further with reference to the judgment by the Court of Justice of the European Union^74^ in *TK v Asociaţia de Proprietari bloc M5A-ScaraA* that the interest of the controller must be present and effective as at the date of the data processing.^75^ This means that any future collaboration the data-providing scientist may expect to materialize at a later time cannot be relied upon. Even the possibility to be cited may provide too weak an argument. However, the data-providing scientist can also rely on the interest of the recipient as Article 6(1)(f) states that the legitimate interest may be that of a third party. As the data-providing scientist’s organization is the sole controller for the disclosure,^76^ the recipient scientist is such a third party whose legitimate interest is demonstrated through the existence of the research project pursued by the third party. Nevertheless, to rely on Article 6(1)(f), the initial controller still needs to perform the balancing test, having to demonstrate that the disclosure meets the reasonable expectations of the data subject. This obligation goes along with the transparency requirements of Article 13(3) that any further processing must be communicated to the data subject with clear information on the right to object.

## VIII. DISCUSSION OF RESULTS IN THE CONTEXT OF THE CHANGING EU LEGISLATIVE FRAMEWORK

We have demonstrated how correctly specifying purposes and mapping them to the processing operations helps the controller identify a suitable GDPR legal basis for the processing of personal data. The case of the research database in Italy referenced above clearly highlights that it is not always easy for the controller to correctly delineate purposes or indeed recognize micro-level purposes that, without an explicit link to an overarching macro-purpose, do not allow the controller to demonstrate compliance with the GDPR, including the principle of lawfulness. This separation is all the more important if the controller wants to rely on consent as a legal basis or base the processing on a legislation. In relation to the latter, that is, legal bases under Articles 6(1)(c) or 6(1)(e), the landscape is constantly changing both on the national and EU levels, especially in terms of secondary use of personal data. While the principles remain, the underlying national and EU legislation that controllers can cite when relying on Articles 6(1)(c) or 6(1)(e) is still taking shape.

The newer EU Regulation 2022/868, known as the Data Governance Act (DGA), provides a framework for secondary use of protected data, including personal data. The secondary uses within the scope of the DGA include scientific research.^77^ However, as the EDPB and the European Data Protection Supervisor (EDPS) point out in their Joint Opinion 03/2021 (paragraph 56), the DGA does not provide sufficiently for purposes and tasks assigned to stakeholders involved in the secondary use and therefore does not provide a legal basis under Article 6(1) of the GDPR.^78^ The creation of a legal basis for the processing of personal data was indeed not an intention of the legislator, as has been clarified in the preamble of the DGA (Recital 4). This is left to the legislators on the level of the EU Member States, as envisaged under the GDPR, as well as applicable sector-specific legislation on the EU level.

In this respect, a particularly relevant EU legislation is the proposed EU Regulation for a European Health Data Space.^79^ The current draft of the EHDS Regulation, as of the time of writing, states in the Impact Assessment section that one objective is to offer an alternative to consent as the legal basis for processing personal data in the context of secondary use. A list of a permitted scope of purposes for secondary use is provided. Where users can legitimately request access for one of these purposes, the draft EHDS Regulation creates a legal obligation for certain controllers, known as ‘data holders’, to disclose electronic health data to ‘health data access bodies’ that act as permit authorities. The draft legislation further provides a legal basis as a task in the public interest for the health data access bodies to process the data to the extent necessary for the individual data access request and to disclose the data in a secure processing environment, also mandated to the health data access bodies. Under certain circumstances, data holders are foreseen to also disclose electronic health data to users applying for access directly.

The aim of the EHDS is to provide a mechanism for secondary use of health data for research and to overcome some of the identified challenges in establishing a valid legal basis. However, the current draft only provides legal bases for a *limited* set of relevant processing activities because most of the processing is related directly to the data access request. The only data processing independent of a data access request is the characterization of data for data catalogues and the classification of data according to quality labels, which still need to be further defined and which may or may not require the processing of personal data. Any processing of personal health data outside the data access request within the EHDS framework still needs its own legal basis. This means that a careful analysis of the processing activities taking place around research and secondary use and their mapping on either the EHDS framework or indeed a purpose not foreseen in the EHDS Regulation will still be required in the future. This will be particularly relevant for pan-European research infrastructures as envisaged, for example, in the area of genetic data and health data through the European ‘1+ Million Genomes Initiative’, a joint declaration signed by most of the EU Member States as well as Norway and the UK in a collaborative manner, where existing data will be harmonized across Europe and made available for secondary use according to a common data governance framework.^80^

Even within the EHDS, there may be limitations for the scientific research use of data, particularly as regards to preserving data from a study to ensure reproducibility or to enable reuse. Article 46(9) of the proposed EHDS Regulation states that a ‘data permit shall be issued for the duration necessary to fulfil the requested purposes which shall not exceed 5 years’. The permit can be extended once, with the extension also limited to 5 years maximum. The proposed rule is that data must be deleted from the secure processing environment within 6 months after the expiry of the data permit. The duration of the permit is intended to provide sufficient time for the user to complete an analysis, but not to preserve the data for reproducibility. However, in research, it is not possible to define the purpose such that it fits into a certain time frame. Rather, as we described above, the time frame during which the data needs to be processed will vary across research projects depending on the nature of their specific purpose(s). An option is given by the EHDS Regulation to store formulas under which data were processed and pre-processed, but the main element, the underlying data, are not held and the data holder who originally provided the data has no legal basis to keep these data longer than needed for its own purposes. In short, the proposed EHDS Regulation does not appear to give sufficient consideration to study data preservation and reproducibility. One could therefore question if a researcher can pursue scientific research in the EHDS that is in keeping with the ethical and methodological standards of biomedical research.

The approach to limit data processing to the user’s purposes and not to keep data beyond the duration of a data permit also limits opportunities for re-use of ‘enriched data’. These are data that have been transformed eg from unstructured into structured data and/or have been cleaned for the purposes of secondary use, generated only in the context of a defined purpose for data use. Subsequently, these enriched data are to be returned to the data holder^81^ who has to decide if these data are valuable or not, and whether the enriched data should be retained.^82^ However, the data holder can only decide to retain data within the scope of its own purposes. As the initial data were generated in a setting fit for the data holder’s own purposes, the data suitable for secondary use purposes will often not meet the needs of the data holder. Where the data holder has no necessity to hold on to the enriched data for its own purposes, the lack of a legal basis means the data must be deleted. The principal shortcoming of this approach is that, for any future requests by other (prospective) data users, the steps of structuring and cleaning the data would need to be performed anew, creating undue inefficiencies.

The above-described weaknesses demonstrate that even for legislators, it is relevant to expend efforts to ensure that sector-specific legislative proposals define purposes of processing personal data in a manner that is consistent with the GDPR requirements for purpose specification.

## IX. CONCLUSION

The purpose behind data processing has a central role in many of the GDPR's articles. Yet, its conceptual underpinnings and practical implications remain under-explored in both regulatory guidance and academic literature. Seeking to fill these lacunae, in this article, we have argued that an adequate determination of the purpose requires building on the relevant data protection principles of Article 5, as well the related concrete compliance obligations under various articles of the GDPR. Based on this approach, we provide actionable guidance designed to help researchers correctly specify purposes for the intended processing of personal data. This exercise serves as both an accountability tool and a rules-based methodology for specifying purposes in a manner that will simplify the controller’s subsequent GDPR compliance. We also show that in the research context, the full and precise purpose is not always obvious; controllers are prone to confusing the overall interest in the processing with the processing-related outcome. In multi-stage processing environments involving long chains of processing, there is also a danger that micro-level purposes may be misconstrued as distinct purposes, while in reality they serve the same overarching purpose. On the other hand, researchers may fail to ascertain that processing operations are performed for distinct purposes, giving rise to different non-compliance risks.

Purpose specification is further important for defining the correct legal basis for the processing of personal data. As discussed in this article, in order to establish a valid legal basis, controllers must correctly specify the purpose(s) for which they intend to process personal data. Illustrative examples of this dependency are legal obligations that are associated with research such as pseudonymization of research data or reporting requirements in clinical trials. Such obligations fall under a different legal basis, Article 6(1)(c), rather than the processing for the actual primary purposes that led to the data collection by the researcher. We also show by way of a recent example that where consent, reflected in Article 6(1)(a), is the legal basis for processing personal data, and it fails to correctly link micro-purposes with the macro-purpose, it may jeopardize the researcher’s ability to lawfully use the data for the macro-purpose. This will likely be the case where the researcher has obtained the data subject’s explicit consent for the micro-purpose only, rendering the consent invalid for the overall (macro-level) purposes the researcher intends to pursue.

The necessity to frame the relevant purposes for the processing is not only relevant to data controllers, but also extends to any legislator that seeks to create a legal basis for the processing of personal data. As illustrated by the legislative process around the proposed EHDS Regulation, without giving purpose specification a careful consideration, the proposed legislation may fail to describe in sufficient detail the envisaged processing activities necessary for performing a task in the public interest, ie to comply with Articles 6(1)(e) and 9(2) of the GDPR. The full scope of the processing must be reflected in the eventually finalized version of the EHDS Regulation, as otherwise controllers may be left in a situation that impedes their ability to perform their mission under that law, or that intended objectives cannot be achieved.

It is worth highlighting that the establishment of a valid legal basis is but one obligation incumbent upon the controller during the phase of designing data processing. Both purpose specification and identification of the legal basis are necessary for enabling the controller as a first step to subsequently comply with other GDPR compliance obligations, including, for example: complying with transparency requirements vis-à-vis the data subject, demonstrating fairness of processing, as well as respecting the principles of data minimization and storage limitation. As shown in this article, some of the specific compliance requirements vary depending on the selected legal basis. For example, relying on consent or the legitimate interest requires the controller to take additional steps to comply with the transparency and fairness obligations.

Correctly framing the purpose(s) of processing personal data under the GDPR requires domain expertise in order to delineate and accurately describe the envisaged achievements. The controller must also have a good understanding of the processing chain and be aware of the applicable legal framework under which the controller operates. Therefore, an interdisciplinary cooperation between scientific domain experts, data scientists, and legal experts is highly recommended for correctly specifying the purposes and the associated GDPR legal bases. This cooperation is particularly important during the design phase of a scientific research project, that is, prior to initiating data processing. In this manner, the controller can comprehensively map out its GDPR compliance obligations throughout the envisaged data lifecycle, including any data processing activities occurring downstream. Having correctly defined the purposes of processing and their associated GDPR legal bases is a vital precondition to enabling this overall compliance.

## Footnotes

1 GDPR Recital 2.

2 GDPR Recitals 1–5.

3 GDPR Art. 1(3).

4 The European Data Protection Board, *Guidelines 07/2020 on the Concepts of Controller and Processor in the GDPR*, 7 July 2021. https://edpb.europa.eu/our-work-tools/documents/public-consultations/2020/guidelines-072020-concepts-controller-and_en.

5 GDPR Art. 5(2). The principles under GDPR Art. 5(1) are: lawfulness, fairness and transparency; purpose limitation; data minimization; accuracy; storage limitation; and integrity and confidentiality.

6 Karen Yeung & Lee A. Bygrave, *Demystifying the Modernized European Data Protection Regime: Cross-disciplinary Insights from Legal and Regulatory Governance Scholarship,* 16 Regul. Gov. 137 (2022).

7 Sean Sirur, Jason R.C. Nurse & Helena Webb, *Are We There Yet?: Understanding the Challenges Faced in Complying with the General Data Protection Regulation (GDPR)*, in Proceedings of the 2nd International Workshop on Multimedia Privacy and Security. Presented at the CCS ’18: 2018 ACM SIGSAC Conference on Computer and Communications Security, Toronto Canada: ACM 88 (2018); Christopher F. Mondschein & Cosimo Monda, *The EU’s General Data Protection Regulation (GDPR) in a Research Context’*, in Fundamentals of Clinical Data Science, ed. by Pieter Kubben, Michel Dumontier, & Andre Dekker (Cham: Springer International Publishing) 55 (2019).

8 Marlene Saemann and others., *Investigating GDPR Fines in the Light of Data Flows*, 4 Proceedings on Privacy Enhancing Technologies 314 (2022).

9 Mondschein & Monda, *supra* note 7.

10 Regina Becker & others, *Applying GDPR Roles and Responsibilities to Scientific Data Sharing*, 12 INT. Data Priv. Law 207 (2022).

11 Alison Cool, *Impossible, Unknowable, Accountable: Dramas and Dilemmas of Data Law*, 49 SOC. STUD. SCI. 503 (2019); Maria Christofidou, Nathan Lea & Pascal Coorevits, *A Literature Review on the GDPR, COVID-19 and the Ethical Considerations of Data Protection During a Time of Crisis*, 30 Yearb. Med. Inform. 226 (2021).

12 Giovanni Comandé & Giulia Schneider, *It’s Time: Leveraging the GDPR to Shift the Balance towards Research-Friendly EU Data Spaces*, 59 Common Mark. Law Rev. 739 (2022); Giovanni Comandè & Giulia Schneider, *Can the GDPR Make Data Flow for Research Easier? Yes It Can, by Differentiating! A Careful Reading of the GDPR Shows How EU Data Protection Law Leaves Open Some Significant Flexibilities for Data Protection-Sound Research Activities* 41 Comput. Law Security Rev. (2021); Giulia Schneider & Giovanni Comandé, *Differential Data Protection Regimes in Data-Driven Research: Why the GDPR Is More Research-Friendly Than You Think* (Rochester, NY: Social Science Research Network, July 14, 2021).

13 GDPR Art. 5(1)(b).

14 Heidi Beate Bentzen, *Context as Key: The Protection of Personal Integrity by Means of the Purpose Limitation Principle*, in Research Handbook on EU Data Protection Law, ed. by Eleni Kosta, Ronald Leenes & Irene Kamara; Edward Elgar Publishing (2022).

15 Regina Becker and others, *Secondary Use of Personal Health Data: When Is It ‘Further Processing’ Under the GDPR, and What Are the Implications for Data Controllers?*, Eur. J. Health Law 1 (2022).

16 Beate Bentzen, *supra* note 14.

17 The European Data Protection Board, *Guidelines 4/2019 on Article 25 Data Protection by Design and by Default Version 2.0*, Oct. 20, 2020.

18 The European Data Protection Board, *Guidelines 2/2019 on the Processing of Personal Data under Article 6(1)(b) GDPR in the Context of the Provision of Online Services to Data Subjects (Version 2.0)*, Oct. 8, 2019.

19 Olga Tzortzatou-Nanopoulou and others, *Secondary Use Of Data For Research Across Europe: In Search Of A Minimal Common Denominator* (Forthcoming); Johan Hansen and others, *Assessment of the EU Member States’ Rules on Health Data in the Light of GDPR* (Luxembourg: Publications Office of the European Union, 2021).

20 Max Von Grafenstein, Timo Jakobi & Gunnar Stevens, *Effective Data Protection by Design through Interdisciplinary Research Methods: The Example of Effective Purpose Specification by Applying User-Centred UX-Design Methods*, 46 Comput. Law Security Rev (2022).

21
*Ibid.*

22 GDPR Art. 5(1)(d).

23 The European Data Protection Board, *supra* note 17.

24 GDPR Art. 5(1)(f).

25 GDPR Art. 30(1).

26 The European Data Protection Board, *supra* note 4.

27 Becker and others, *supra* note 10.

28 Becker and others, *supra* note 15.

29 The European Data Protection Board, *supra* note 17.

30 Article 29 Data Protection Working Party, *Opinion 03/2013 on Purpose Limitation*, Apr. 2, 2013. (NB: this opinion concerns the DPD, not the GDPR).

31 Imane Fouad and others, *On Compliance of Cookie Purposes with the Purpose Specification Principle*, in 2020 IEEE European Symposium on Security and Privacy Workshops (EuroS&PW), Presented at the 2020 IEEE European Symposium on Security and Privacy Workshops (EuroS&PW), Genoa, Italy: IEEE, 326 (2020); Michele Finck & Asia J. Biega, *Reviving Purpose Limitation and Data Minimisation in Data-Driven Systems*, Technology and Regulation 44 (2021); Von Grafenstein, Jakobi & Stevens, *supra* note 20.

32 Fouad and others, *supra* note 31.

33 See, for example, GDPR Art. 6(1)(a) and Art. 25(2).

34 For example, GDPR Art. 5(1)(b).

35 GDPR Art. 5(1)(b), 25(2), and 35(7).

36 GDPR Art. 5(1)(d).

37 GDPR Art. 5(1)(e), 25(2), 13(2), and 14(2).

38 GDPR Art. 5(1)(c), 25(2), and 35(7)(b).

39 GDPR Art. 13(1–2), and 14(1–2); these articles demonstrate consistency: they require the purpose to be specified separately from the means such as the categories of personal data to be processed and the duration of processing.

40 The European Data Protection Board, *supra* note 18.

41 The European Data Protection Board, *supra* note 4.

42 Becker and others, *supra* note 15.

43 GDPR Recital 159.

44 The European Data Protection Board, *Guidelines 05/2020 on Consent under Regulation 2016/679*, May 4, 2020.

45 Where we refer to a ‘researcher’ in the text, the legally responsible entity will always be the researcher’s home institution.

46 European Science Foundation, *Good Scientific Practice in Research and Scholarship.*, ESF Science Policy Briefing, 1 (2000).

47 Becker and others, *supra* note 15.

48 Zornitza Stark and others, *Integrating Genomics into Healthcare: A Global Responsibility*, 104 AM. J. Hum. Genet. 13 (2019).

49 Luigi Coppola and others, *Biobanking in Health Care: Evolution and Future Directions*, 17 J. Transl. Med. 172 (2019).

50 Ilkka Lappalainen and others, *The European Genome-Phenome Archive of Human Data Consented for Biomedical Research*, 47 Nat. Genet. 692 (2015).

51 Kimberly A. Tryka and others, *NCBI’s Database of Genotypes and Phenotypes: DbGaP*, 42 Nucleic Acids Res. D975 (2014).

52 The scenario described here is a representative example. However, other situations may be possible such as a healthcare institution whose mission is limited to healthcare provision, but it responds to the interest of the recipient institution to establish a biobank or a repository. Such a scenario leads to a different purpose definition and correspondingly different associated processing steps.

53 Note that this scenario is different from situations where the initial controller establishes a data collection with an intention to use it for own research in the future. The latter scenario is discussed below, under the Section V.

54 This is an illustrative example of how the controller can specify purposes in a stepwise, iterative manner. The controller starts by identifying a ‘draft’ purpose, which broadly describes the controller’s intention in relation to the processing of the personal data (eg making the data widely available for external researchers). Initially, the draft purpose lacks the specification required to enable compliance with the GDPR obligations, including determining what processing operations are needed to achieve the purpose, as well as demonstrating compliance with the Art. 5(1) principles of the Regulation. However, once the controller has identified the repository through which it intends to make the data available, the controller will have more information to further specify and (in the event of processing personal data for more than one specific purpose) delineate the purpose(s).

55 ‘Purpose’ in this case does not refer to the notion of purpose as used in the GDPR.

56 Council for International Organizations of Medical Sciences (CIOMS), *International Ethical Guidelines for Health-Related Research Involving Humans* (2016).

57 GDPR Recital 33.

58 Garante per la Protezione dei dati Personali, *Parere Ai Sensi Del Ai Sensi Dell’art. 110 Del Codice e Dell’art. 36 Del Regolamento*, 30 June 2022. https://www.garanteprivacy.it/web/guest/home/docweb/-/docweb-display/docweb/9791886 (accessed May 9, 2023).

59 Saemann and others, *supra* note 8.

60 Becker and others, *supra* note 15.

61 Article 29 Data Protection Working Party, *Opinion 06/2014 on the Notion of Legitimate Interests of the Data Controller under Article 7 of Directive 95/46/EC*, 9 April 2014. (NB: this opinion concerns the DPD, not the GDPR).

62 As per GDPR Art. 6(3) and GDPR Recital 45.

63 European Data Protection Board, *Opinion 3/2019 Concerning the Questions and Answers on the Interplay between the Clinical Trials Regulation (CTR) and the General Data Protection Regulation (GDPR)*, Jan. 23, 2019.

64 The European Data Protection Board, *supra* note 44.

65 European Science Foundation, *supra* note 46.

66
*Ibid.*

67 CIOMS, *supra* note 56.

68 However, the obligation to inform the data subject about this processing, even if without obtaining a separate explicit consent, still applies, in accordance with the transparency and fairness principles of the GDPR. This reflects that the purpose must allow to assess which processing is or is not included in the processing and that data subjects should never be surprized about how their data are processed. (See The European Data Protection Board, *supra* note 17; Article 29 Data Protection Working Party, *Guidelines on transparency under Regulation 2016/679,* Nov. 29, 2017).

69 For example, the Norwegian Health Registries Act gives public health registries a mandate to prepare anonymous or anonymized data. See *Lov om helseregistre og behandling av helseopplysninger (helseregisterloven*; Article 19 (LOV-2014-06-20-43).

70 Article 29 Data Protection Working Party, *supra* note 61.

71 GDPR Recital 32; The European Data Protection Board, *supra* note 44 (Para 58).

72 Becker and others, *supra* note 15.

73 Article 29 Data Protection Working Party, *supra* note 61.

74 Court of Justice of the European Union (Third Chamber), *TK v Asociatia de Proprietari bloc M5A-ScaraA*, (2019). https://curia.europa.eu/juris/document/document.jsf;jsessionid=4E947E507B17BF2713FC28BF8214DEB0?text=&docid=221465&pageIndex=0&doclang=en&mode=lst&dir=&occ=first&part=1&cid=20627 (accessed May 9, 2023).

75 The European Data Protection Board, *Recommendations 02/2021 on the Legal Basis for the Storage of Credit Card Data for the Sole Purpose of Facilitating Further Online Transactions*, May 19, 2021.

76 Becker and others, *supra* note 10.

77 European Parliament and the Council, *Regulation (EU) 2022/868 of the European Parliament and of the Council of 30 May 2022 on European Data Governance and Amending Regulation (EU) 2018/1724 (Data Governance Act)*, 2022.

78 The European Data Protection Board and The European Data Protection Supervisor, *EDPB-EDPS Joint Opinion 03/2021 on the Proposal for a Regulation of the European Parliament and of the Council on European Data Governance (Data Governance Act) Version 1.1*, 9 June 2021. https://edpb.europa.eu/system/files/2021-03/edpb-edps_joint_opinion_dga_en.pdf (accessed May 9, 2023).

79 The European Parliament and The Council, *Proposal for a Regulation of the European Parliament and of the Council on the European Health Data Space*, 2022. https://health.ec.europa.eu/publications/proposal-regulation-european-health-data-space_en (accessed May 9, 2023).

80 ‘The European “1+ Million Genomes” Initiative’. https://digital-strategy.ec.europa.eu/en/policies/1-million-genomes (accessed May 9, 2023).

81 Art. 37(1)(p) EHDS Regulation, draft of May 3, 2022.

82 Art. 41(5) EHDS Regulation, draft of May 3, 2022.

